# Association between oxygen saturation level during bronchoscopy and post-bronchoscopy adverse events: a retrospective cohort study

**DOI:** 10.1186/s12931-022-02063-0

**Published:** 2022-06-02

**Authors:** So Yeon Kim, Hyo Jin Lee, Jung Kyu Lee, Tae Yeon Park, Eun Young Heo, Deog Kyeom Kim, Hee Soon Chung, Hyun Woo Lee

**Affiliations:** Division of Pulmonary and Critical Care Medicine, Department of Internal Medicine, Seoul Metropolitan Government-Seoul National University Boramae Medical Center, 20, Boramae-ro 5-gil, Dongjak-gu, Seoul, 07061 Republic of Korea

**Keywords:** Bronchoscopy, Oxygen saturation, Complications, Safety

## Abstract

**Background:**

Flexible bronchoscopy is widely used to diagnose and treat various respiratory diseases. However, caution is warranted for post-bronchoscopy adverse events. Although desaturation frequently occurs during bronchoscopy, its clinical impact and the optimal oxygen saturation level during the procedure remain unclear. This study aimed to investigate whether the percutaneous oxygen saturation (SpO_2_) level during bronchoscopy is associated with the development of post-bronchoscopy respiratory adverse events.

**Methods:**

In this single-center retrospective cohort study conducted from March 2020 to February 2021, 569 patients were classified into high or low oxygen saturation groups based on the SpO_2_ level during bronchoscopy. The primary outcome was post-bronchoscopy respiratory adverse events, and secondary outcomes were other post-bronchoscopy adverse events and clinical outcomes.

**Results:**

Among 569 patients, 458 and 111 patients were classified into the high oxygen saturation (SpO_2_ > 96%) and low oxygen saturation (SpO_2_ ≤ 94%) groups, respectively. After propensity score matching, the low oxygen saturation group had more post-bronchoscopy respiratory and febrile adverse events than the high oxygen saturation group. In the multivariable regression analysis, low SpO_2_ level during bronchoscopy was an independent risk factor for post-bronchoscopy respiratory adverse events (odds ratio = 3.16 [95% confidence interval 1.37–7.30]). In the low oxygen saturation group, the high-risk subgroups for post-bronchoscopy respiratory adverse events were the elderly, women, current smokers, and patients with chronic obstructive pulmonary disease or acute decompensated heart failure before bronchoscopy. There was no significant difference in the length of hospital stay, intensive care unit admission, or mortality between the high and low oxygen saturation groups.

**Conclusions:**

Close monitoring is recommended for patients with SpO_2_ ≤ 94% during bronchoscopy due to the increased risk of respiratory adverse events after the procedure.

**Supplementary Information:**

The online version contains supplementary material available at 10.1186/s12931-022-02063-0.

## Background

Flexible fiberoptic bronchoscopy (FB) is a safe and effective procedure used to diagnose and treat diverse respiratory diseases [1]. Significant oxygen desaturation events during FB have been reported to range from 1 to 97% in different study settings [2–4]. During bronchoscopy, the arterial partial pressure of oxygen may decrease by over 10–20 mmHg, increasing the risk of respiratory failure [5, 6]. Risk factors related to desaturation during FB include lung function, comorbid diseases, use of a sedative, and procedure-related factors [7–9]. Various oxygen supplementation strategies have been proposed to prevent desaturation during FB. These include conventional oxygen therapy (COT), high flow nasal cannula (HFNC), continuous positive airway pressure (CPAP), and noninvasive ventilation (NIV) [5]. Monitoring the oxygen saturation level with percutaneous pulse oximetry is recommended to assess and manage significant desaturation events [1]. However, the optimal oxygen saturation range during FB remains unclear. Few studies have investigated the optimal oxygen saturation level during FB because hypoxemic events usually resolve instantly after oxygen supplementation and rarely cause complications [8]. Nonetheless, recovery from the cardiopulmonary distress caused by FB may take minutes to hours, depending on the patients’ lung function [10]. Therefore, a high or low maintenance range of oxygen saturation level may worsen clinical outcomes, and oxygen saturation level during FB may be a potential predictor of prognosis [11].

There is limited evidence to support the association between the risk of post-bronchoscopy adverse events and the oxygen saturation level during FB. Many patients who present with hypoxemia (arterial oxygen pressure < 60 mmHg) during bronchoscopy have shown significant changes in physiologic parameters related to cardiac function. However, there has been no significant increase in the incidence of cardiac arrhythmia among post-bronchoscopy patients [12–14]. Conversely, hypoxemia at the end of bronchoscopy was significantly correlated with the development of new-onset major cardiac arrhythmia [15]. A high fraction of inspired oxygen during bronchoscopy in critically ill patients did not alter the risk of post-bronchoscopy intubation [16].

We aimed to compare the post-bronchoscopy adverse events and clinical outcomes between patients with high and low oxygen saturation levels during FB.

## Methods

Our study is in accordance with the Strengthening the Reporting of Observational Studies in Epidemiology (STROBE) guidelines [17].

### Study design and eligibility criteria

We conducted a retrospective cohort study via electronic medical record review at Seoul National University Boramae Medical Center in Korea from March 1, 2020, to February 28, 2021. We screened adult patients aged ≥ 18 years who underwent FB with continuous percutaneous oxygen saturation (SpO_2_) monitoring on an inpatient and outpatient basis. Following our hospital policy, FB was only performed on patients with a negative nasopharyngeal polymerase chain reaction test result for coronavirus disease 2019 within the previous 48 h. The inclusion criteria were: (1) patients suspected of respiratory disease based on chest computed tomography and (2) patients with pre-bronchoscopy SpO_2_ maintained above 94% with or without oxygen supplementation. The exclusion criteria were: (1) patients who were unclassifiable into high oxygen or low oxygen saturation groups due to severely fluctuating SpO_2_, (2) patients with SpO_2_ of mainly 95%, (3) patients who were unable to complete FB due to prolonged (> 1 min) severe hypoxemia (SpO_2_ < 90%) despite maximal oxygen supplementation, (4) patients admitted to the intensive care unit (ICU) while performing FB, (5) patients with recent (within 6 weeks) acute myocardial infarction and acute stroke, (6) pregnant patients, (7) patients with life expectancies of < 1 month, and (8) immunocompromised patients (e.g., patients undergoing chemotherapy for malignancy). Severely fluctuating SpO_2_ was defined as SpO_2_ levels spanning both high and low oxygen saturation ranges for more than 1 min. Patients in the low (SpO_2_ ≤ 94%) and high (SpO_2_ > 96%) oxygen saturation group were allowed SpO_2_ > 96% and SpO_2_ ≤ 94% for less than 1 min, respectively.

### Clinical indications of bronchoscopy

FB was performed for both diagnostic and therapeutic purposes. FB aided in the diagnosis of patients with respiratory symptoms (e.g., persistent cough, hemoptysis, wheezing), radiologic abnormalities (e.g., lung parenchymal infiltration, broncho-pleural fistula, atelectasis, pleural effusion, mass), and clinical suspicion of pneumonia, malignancy, mycobacterial infection, abscess, interstitial lung disease, or any endobronchial lesion. Bronchoscopic toileting or bronchoalveolar lavage (BAL) was also implemented to remove impacted secretions and reverse atelectasis.

### Standard procedure for bronchoscopy

The patients’ percutaneous oxygen saturation, heart rate, respiratory rate, blood pressure, and cardiac rhythm were monitored upon arrival in the bronchoscopy room using patient monitors with a finger pulse oximeter and blood pressure, and electrocardiography (ECG) monitoring capabilities. Patients were monitored full-time until they left the bronchoscopy room. We used identical IntelliVue MP5 (Philips) monitoring devices with the same finger pulse oximeters for all patients*.* The respiratory physician performing the FB decided whether each patient required a low to moderate dose of midazolam for sedation. In supine position without head elevation, FB was performed trans-nasally or trans-orally. We used a 2% lidocaine solution for local anesthesia of the vocal cord and bronchial tree. Chest radiography was routinely performed in patients who had undergone transbronchial biopsy.

### Monitoring oxygen saturation (SpO_2_) during bronchoscopy

Each session was attended by two experienced physicians, a nurse, and a technician. As one physician performed the FB, the other physician monitored the oxygen saturation. Supplemental oxygen was administered at the physicians’ discretion, and oxygen delivery was not routinely provided. As per our in-hospital protocol, the procedure was immediately stopped when the SpO_2_ level dropped below 90% or by more than 4% from baseline. Supplemental oxygen would subsequently be administered until the SpO_2_ level recovered. The quantity and modality of oxygen supplementation were decided based on the severity of desaturation and the patients’ overall condition. Primarily, a nasal prong was applied for oxygen delivery beginning at 2–3 L/min, and the flow rate was adjusted according to the SpO_2_ level. If desaturation is refractory, a simple or non-rebreather facial mask was applied at 6–15 L/min. The SpO_2_ level was automatically assessed every minute, and the highest and lowest levels were recorded.

### Study group definition based on SpO_2_ level

Eligible patients were classified into the low or high oxygen saturation groups according to the SpO_2_ level detected by percutaneous pulse oximetry during FB. The low oxygen saturation group included patients who maintained SpO_2_ between 90 and 94% for most of the duration of FB. The high oxygen saturation group included patients who maintained SpO_2_ above 96% for most of the duration of bronchoscopy.

### Variables and outcomes

We collected the patients’ clinical information, including age, body mass index (BMI), smoking status, comorbidities (hypertension, diabetes mellitus, congestive heart failure, arrhythmia, chronic kidney disease, chronic liver disease, connective tissue disease, dementia, and history of other malignancies), and respiratory diseases (chronic obstructive pulmonary disease [COPD], bronchiectasis, asthma, interstitial lung disease, tuberculosis destroyed lung, history of lung cancer, lung resection, or radiation therapy). We also investigated the symptoms and pulmonary function test results within the past year. Clinical diagnoses, acute comorbidities (acute decompensated heart failure, acute coronary syndrome, pneumothorax, and pleural effusion), and surrogates for respiratory failure (desaturation event, oxygen demand, SpO_2_:FiO_2_ [SF ratio], and respiratory rate-oxygenation [ROX] index) were assessed before FB.

The primary outcome was post-bronchoscopy respiratory adverse events. The secondary outcomes were (1) other post-bronchoscopy adverse events, including febrile, hemodynamic, cardiac, and cerebrovascular events, and (2) clinical outcomes after FB, including ICU admission, length of hospital stay, and all-cause mortality within 7 days. Post-bronchoscopy adverse events were defined as unexpected medical occurrences in a patient who received FB, which did not necessarily have a causal relationship [18]. We analyzed all types of post-bronchoscopy adverse events that (1) occurred within 72 h after FB, (2) were explainable by the physiologic changes after FB, (3) have repeatedly been reported in previous studies, and (4) were determined by the physicians’ assessment of causality. Post-bronchoscopy respiratory adverse events were defined as a composite outcome, including pneumonia, atelectasis, respiratory failure, pneumothorax, bronchospasm, and acute exacerbation of an underlying chronic respiratory disease.

### Statistical analyses

The chi-squared test or Fisher’s exact test was used for the binary variables. The Student’s t-test or Wilcoxon rank-sum test was used for continuous variables. The study population was matched at a two-to-one ratio based on the propensity scores calculated using all covariates in the baseline clinical features. All measured baseline covariates were included in the model, and two-to-one propensity score matching was implemented to improve the precision while minimizing the bias [19–21]. Standardized differences were used to compare the baseline characteristics and clinical features in the propensity score-matched populations [19]. According to Cohen’s effect size index for the comparison of two sample means, standardized differences of 0.2, 0.5, and 0.8 represent small, medium, and large imbalances in the baseline covariates [22].

Independent risk factors for post-bronchoscopy respiratory adverse events were identified through univariable and multivariable regression analyses using the best subset selection method. A variance inflation factor > 4.0 was considered at risk for significant multicollinearity. The association between oxygen saturation level and post-bronchoscopy respiratory adverse events was evaluated in multiple subgroups classified by clinically important factors. P-value < 0.05 was considered significant. Statistical analyses were performed using the R statistical software version 4.1.0 (R Core Team [2020], Vienna, Austria).

## Results

**Fig. 1:**
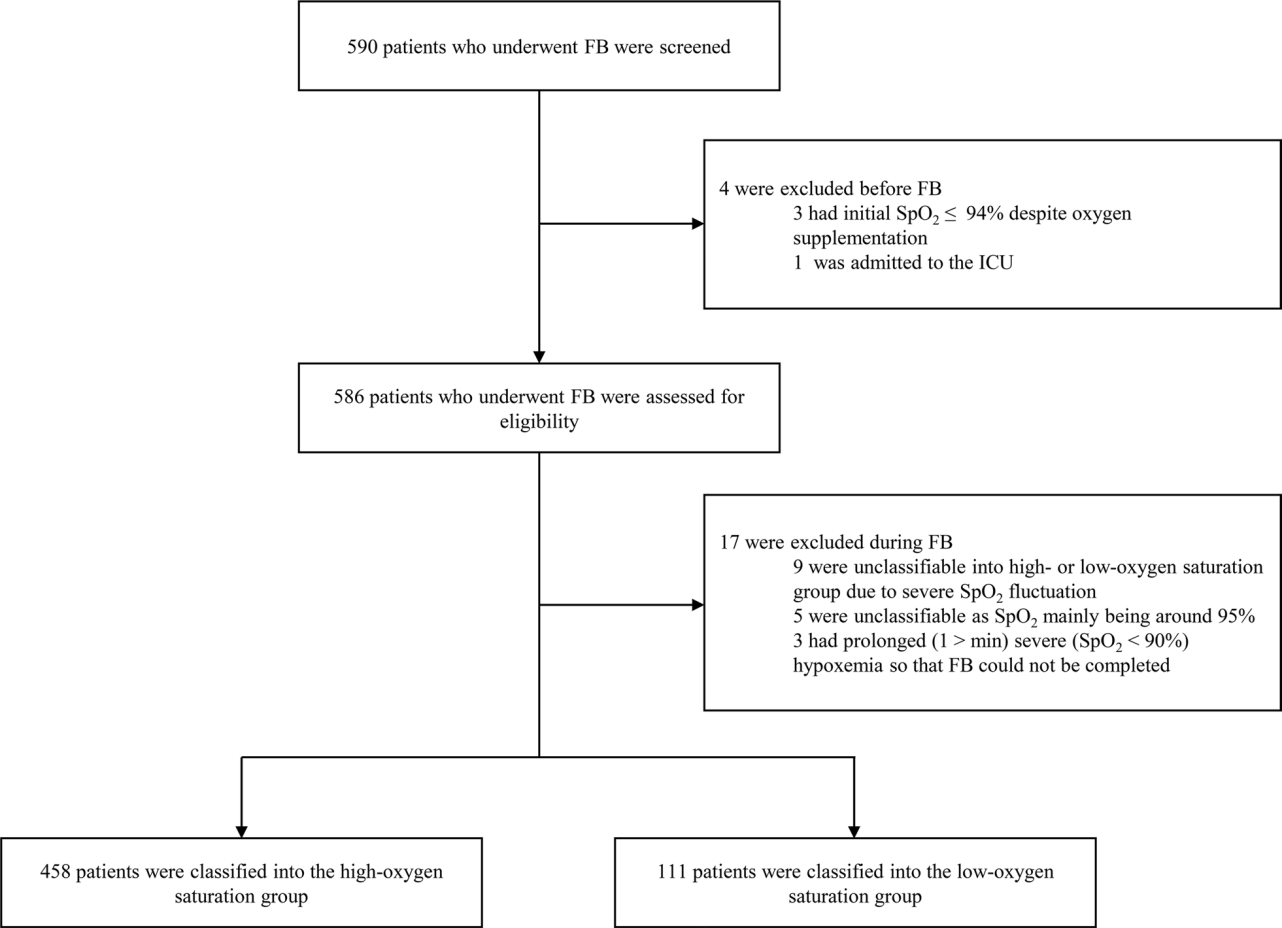
Flowchart of screening, eligibility assessment, and classification of the study population. *FB* flexible fiberoptic bronchoscopy; *SpO*_2_ saturation of percutaneous oxygen; *ICU* intensive care unit

We screened 590 patients who underwent FB. Twenty-one patients were excluded due to initial saturation ≤ 94% despite oxygen supplementation (n = 3), ICU admission during FB (n = 1), inability to classify patients into either group due to SpO_2_ fluctuations (n = 9), SpO_2_ of mainly 95% (n = 5), and inability to complete FB because of severe persistent hypoxemia (n = 3). A total of 569 patients met the inclusion criteria and were classified into the high oxygen saturation group (n = 458) and low oxygen saturation group (n = 111) (Fig. 1). Compared to the low oxygen saturation group, more patients in the high oxygen saturation group underwent FB on an outpatient basis (27.9% vs*.* 38.4%, P = 0.051), and the median duration of FB was shorter (5 [interquartile range, IQR = 2–16] min vs*.* 7 [IQR = 2–31] min, p < 0.001) in the high oxygen saturation group than in the low oxygen saturation group.

### Baseline characteristics and clinical features

The baseline characteristics of the high and low oxygen saturation groups are summarized in Table 1. The mean age of the patients was 67 years in both groups. Overall, 146 (31.9%) and 43 patients (38.7%) were female in the high and low oxygen saturation groups, respectively. There were no significant differences in demographic features and the incidence of previous medical conditions between the two groups, except for a higher BMI and a lower proportion of tuberculosis-destroyed lungs in the low oxygen saturation group. The most common respiratory diseases found in both groups were bronchiectasis, chronic obstructive pulmonary disease, and tuberculosis destroyed lung. Symptoms and spirometric profiles were similar between the two groups (Table 2). The most common primary indications for FB were the clinical suspicion of mycobacterial infection or pneumonia*.* More patients in the high oxygen saturation group were clinically suspected of atypical pneumonia. After propensity score matching, the statistical differences in baseline characteristics and clinical features disappeared.

**Table 1:** Baseline characteristics of total and propensity score-matched patients

	Total population			Propensity score-matched population		
	High oxygen saturation group (n = 458)	Low oxygen saturation group (n = 111)	P-value	High oxygen saturation group (n = 222)	Low oxygen saturation group (n = 111)	Standardized differences
Age, yr, mean (± SD)	66.9 (13.9)	67.0 (14.7)	0.963	66.4 (15.2)	67.0 (14.7)	0.038
Female, n (%)	146 (31.9)	43 (38.7)	0.206	80 (36.0)	43 (38.7)	0.056
Body mass index, mean (± SD)	21.3 (4.2)	22.4 (4.2)	0.013	22.0 (4.7)	22.4 (4.2)	0.087
Smoking status						
Current smoker, n (%)	86 (18.8)	18 (16.2)	0.624	38 (17.1)	18 (16.2)	0.024
Ex-smoker, n (%)	124 (27.1)	25 (22.5)	0.391	52 (23.4)	25 (22.5)	0.021
Never smoker, n (%)	240 (52.4)	68 (61.3)	0.115	129 (58.1)	68 (61.3)	0.064
PYs, median (IQR)	0 (0–30)	0 (0–20)	0.120	0 (0–20)	0 (0–20)	0.015
Comorbidities, n (%)						
Hypertension	182 (39.7)	43 (38.7)	0.932	86 (38.7)	43 (38.7)	< 0.001
Diabetes mellitus	128 (27.9)	24 (21.6)	0.218	51 (23.0)	24 (21.6)	0.032
Congestive heart failure	51 (11.1)	19 (17.1)	0.119	32 (14.4)	19 (17.1)	0.074
Arrhythmia	19 (4.1)	5 (4.5)	1.000	11 (5.0)	5 (4.5)	0.021
Cerebrovascular accident	88 (19.2)	15 (13.5)	0.207	32 (14.4)	15 (13.5)	0.026
Chronic kidney disease	32 (7.0)	10 (9.0)	0.597	13 (5.9)	10 (9.0)	0.120
Chronic liver disease	25 (5.5)	9 (8.1)	0.405	9 (4.1)	9 (8.1)	0.170
Connective tissue disease	13 (2.8)	3 (2.7)	1.000	5 (2.3)	3 (2.7)	0.029
Dementia	43 (9.4)	5 (4.5)	0.141	21 (9.5)	5 (4.5)	0.195
History of other malignancy	80 (17.5)	23 (20.7)	0.508	37 (16.7)	23 (20.7)	0.104
Respiratory disease, n (%)						
Chronic obstructive pulmonary disease	81 (17.7)	14 (12.6)	0.253	28 (12.6)	14 (12.6)	< 0.001
Bronchiectasis	133 (29.0)	28 (25.2)	0.495	54 (24.3)	28 (25.2)	0.021
Asthma	15 (3.3)	3 (2.7)	0.994	10 (4.5)	3 (2.7)	0.096
Interstitial lung disease	13 (2.8)	3 (2.7)	1.000	5 (2.3)	3 (2.7)	0.029
Tuberculosis destroyed lung	84 (18.3)	9 (8.1)	0.013	22 (9.9)	9 (8.1)	0.063
Nontuberculous mycobacteria	14 (3.1)	5 (4.5)	0.640	7 (3.2)	5 (4.5)	0.070
History of lung cancer	35 (7.6)	4 (3.6)	0.193	19 (8.6)	4 (3.6)	0.208
History of lung resection	12 (2.6)	3 (2.7)	1.000	7 (3.2)	3 (2.7)	0.027
History of thoracic radiation therapy	15 (3.3)	1 (0.9)	0.299	7 (3.2)	1 (0.9)	0.160

Data are expressed as mean (± standard deviation), median (IQR), or number (percentage)

*PYs* pack-years; *SD* standard deviation; *IQR* interquartile range

**Table 2:** Clinical features before bronchoscopy in total and propensity score-matched patients

	Total population			Propensity score-matched population		
	High oxygen saturation group (n = 458)	Low oxygen saturation group (n = 111)	P-value	High oxygen saturation group (n = 222)	Low oxygen saturation group (n = 111)	Standardized differences
Symptoms, n (%)						
Chronic bronchitis	79 (17.2)	13 (11.7)	0.201	28 (12.6)	13 (11.7)	0.027
Chronic cough	87 (19.0)	14 (12.6)	0.150	41 (18.5)	14 (12.6)	0.162
Hemoptysis	54 (11.8)	11 (9.9)	0.695	24 (10.8)	11 (9.9)	0.029
Pulmonary function test^a^						
FVC, L, mean (± SD)	2.8 (0.6)	2.8 (0.6)	0.947	2.9 (0.6)	2.8 (0.6)	0.093
FVC, %, mean (± SD)	85.1 (13.5)	87.9 (13.5)	0.054	87.7 (11.5)	87.9 (13.5)	0.013
FEV_1_, L, mean (± SD)	2.06 (0.5)	2.0 (0.4)	0.489	2.11 ± 0.5)	2.0 (0.4)	0.185
FEV_1_, %, mean (± SD)	89.5 (16.7)	91.4 (16.9)	0.283	92.9 (14.7)	91.4 (17.0)	0.097
FEV_1_/FVC, %, mean (± SD)	73.8 (8.9)	72.9 (8.1)	0.338	74.3 (7.6)	72.9 (8.1)	0.175
BDR, %, median (IQR)	2.0 (0–4.0)	2.0 (0–3.0)	0.351	2.0 (0–4.0)	2.0 (0–3.0)	0.068
DL_CO_, %, mean (± SD)	88.6 (29.6)	87.3 (24.8)	0.787	94.4 (26.0)	87.3 (24.8)	0.280
DL_CO_/VA, %, mean (± SD)	86.2 (29.4)	88.6 (18.4)	0.615	92.5 (26.0)	88.6 (18.5)	0.179
Clinically suspected diagnosis, n (%)						
Aspiration pneumonia	120 (26.2)	28 (25.2)	0.929	59 (26.6)	28 (25.2)	0.031
Atypical pneumonia	41 (9.0)	18 (16.2)	0.038	21 (9.5)	18 (16.2)	0.202
Lung malignancy	121 (26.4)	30 (27.0)	0.992	59 (26.6)	30 (27.0)	0.010
Mycobacterial infection	176 (38.4)	35 (31.5)	0.215	81 (36.5)	35 (31.5)	0.105
Lung abscess	21 (4.6)	5 (4.5)	1.000	10 (4.5)	5 (4.5)	< 0.001
Interstitial lung disease	22 (4.8)	7 (6.3)	0.685	13 (5.9)	7 (6.3)	0.019
Endobronchial lesion	111 (24.2)	16 (14.4)	0.036	45 (20.3)	16 (14.4)	0.155
Combined acute medical conditions, n (%)						
Acute decompensated heart failure	35 (7.6)	11 (9.9)	0.554	20 (9.0)	11 (9.9)	0.031
Acute coronary syndrome	4 (3.6)	9 (2.0)	0.495	8 (3.6)	4 (3.6)	< 0.001
Pneumothorax	17 (3.7)	1 (0.9)	0.224	7 (3.2)	1 (0.9)	0.160
Pleural effusion	133 (29.0)	28 (25.2)	0.495	61 (27.5)	28 (25.2)	0.051
Surrogates for respiratory failure						
Desaturation event, n (%)	90 (19.7)	34 (30.6)	0.017	60 (27.0)	34 (30.6)	0.080
Oxygen demand, L/min, median (IQR)	0 (0–0)	0 (0–1.0)	0.699	0 (0–0)	0 (0–1.0)	0.061
SF ratio, mean (± SD)	451.5 (53.6)	426.2 (66.6)	< 0.001	439.5 (68.2)	426.2 (66.6)	0.197
ROX index, mean (± SD)	22.6 (2.8)	21.6 (3.2)	< 0.001	22.1 (3.5)	21.6 (3.2)	0.153

Data are expressed as mean (± standard deviation), median (IQR), or number (percentage)

*FVC* forced vital capacity; *FEV*_1_ forced expiratory volume in one second; *BDR* bronchodilator test; *DL*_*CO*_ diffusion capacity of the lung for carbon monoxide; *DL*_*CO*_*/VA* diffusion capacity of the lung for carbon monoxide per unit alveolar volume; *SD* standard deviation; *IQR* interquartile range

^a^Bronchodilator response was assessed in 342 patients, and DL_CO_ was assessed in 360 patients

### Parameters and procedures during bronchoscopy

Classification into high and low oxygen saturation groups led to significant differences in the initial, highest, and lowest SpO_2_ during FB between the two groups (P < 0.001 for all parameters; Table 3). More desaturation events were observed in the low oxygen saturation group. Although more sedative agents were used and more invasive procedures, such as BAL and endobronchial ultrasound-guided transbronchial needle aspiration, were performed in the low oxygen saturation group, these differences were insignificant after propensity score matching.

**Table 3:** Parameters and procedures during bronchoscopy

	Total population			Propensity score-matched population		
	High oxygen saturation group (n = 458)	Low oxygen saturation group (n = 111)	P-value	High oxygen saturation group (n = 222)	Low oxygen saturation group (n = 111)	P-value
Oxygen saturation during bronchoscopy						
Initial SpO_2_, mean (± SD)	98.9 (1.5)	96.9 (2.9)	< 0.001	98.8 (1.6)	96.9 (2.9)	< 0.001
Highest SpO_2_, mean (± SD)	99.1 (1.3)	94.9 (2.4)	< 0.001	99.0 (1.4)	94.9 (2.4)	< 0.001
Lowest SpO_2_, mean (± SD)	95.4 (5.2)	87.0 (5.8)	< 0.001	94.8 (5.8)	87.0 (5.8)	< 0.001
Desaturation event, n (%)	87 (19.0)	82 (73.9)	< 0.001	46 (20.7)	82 (73.9)	< 0.001
Desaturation duration > 1 min, n, (%)	0 (0.0)	12 (10.8)	< 0.001	0 (0.0)	12 (10.8)	< 0.001
Duration of bronchoscopy in min, median (IQR)	6.0 (5.0–7.0)	6.0 (5.0–12.0)	0.007	6.0 (5.0–8.0)	6.0 (5.0–12.0)	0.137
Duration of desaturation in min, median (IQR)	0 (0–0)	1.0 (0–1.0)	< 0.001	0 (0–0)	1.0 (0–1.0))	< 0.001
Sedation, n (%)	128 (27.9)	49 (44.1)	0.001	97 (43.7)	49 (44.1)	1.000
Procedure type, n (%)						
Bronchial washing	306 (66.8)	65 (58.6)	0.127	142 (64.0)	65 (58.6)	0.402
Toileting	109 (23.8)	24 (21.6)	0.718	52 (23.4)	24 (21.6)	0.817
Bronchoalveolar lavage	29 (6.3)	14 (12.6)	0.041	15 (6.8)	14 (12.6)	0.114
Biopsy	46 (10.0)	10 (9.0)	0.880	21 (9.5)	10 (9.0)	1.000
EBUS-TBNA	37 (8.1)	18 (16.2)	0.015	24 (10.8)	18 (16.2)	0.220
TBLB	1 (0.2)	1 (0.9)	0.844	0 (0.0)	1 (0.9)	0.723
Foreign body removal	15 (3.3)	2 (1.8)	0.612	8 (3.6)	2 (1.8)	0.570
Bronchoscopist			0.311			0.371
Attending physician alone	221 (48.3)	47 (42.3)		107 (48.2)	47 (42.3)	
Fellow physician, supervised by attending physician	237 (51.7)	64 (57.7)		115 (51.8)	64 (57.7)	

Data are expressed as mean (± standard deviation) or median (interquartile range) and number (percentage)

*EBUS-TBNA* endobronchial ultrasound-guided transbronchial needle aspiration; *TBLB* transbronchial lung biopsy; *SD* standard deviation; *IQR* interquartile range

### Post-bronchoscopy adverse events and clinical outcomes

More post-bronchoscopy adverse events were found in the low oxygen saturation group (Table 4). The low oxygen saturation group had more respiratory (P = 0.001) and febrile adverse events (P < 0.001) than the high oxygen saturation group. However, there was no significant difference in the length of hospital stay, ICU admission, or 7-day mortality after FB between the two groups. Even in the propensity score-matched population, respiratory adverse events were more common in the low oxygen saturation group (P = 0.023).

**Table 4:** Post-bronchoscopy adverse events and clinical outcomes

	Total population			Propensity score-matched population		
	High oxygen saturation group (n = 458)	Low oxygen saturation group (n = 111)	P-value	High oxygen saturation group (n = 222)	Low oxygen saturation group (n = 111)	P-value
Number of patients with post-bronchoscopy adverse events, n (%)						
Respiratory events	43 (9.4)	23 (20.7)	0.001	24 (10.8)	23 (20.7)	0.023
Febrile events	48 (10.5)	29 (26.1)	< 0.001	24 (10.8)	29 (26.1)	0.001
Hemodynamic events	33 (7.2)	7 (6.3)	0.900	16 (7.2)	7 (6.3)	0.939
Cardiac events	11 (2.4)	6 (5.4)	0.175	10 (4.5)	6 (5.4)	0.928
Cerebrovascular events	0 (0.0)	1 (0.9)	0.441	0 (0.0)	1 (0.9)	0.723
Any adverse events	103 (22.3)	35 (32.4)	0.021	49 (22.1)	38 (34.2)	0.025
Clinical outcomes						
Hospital length of stay after bronchoscopy, median (IQR)	2 (1–12)	4 (1–10)	0.99	1 (1–14)	2 (1–11)	0.744
ICU admission after bronchoscopy, n (%)	18 (3.9)	9 (8.1)	0.159	8 (3.6)	9 (8.1)	0.135
7-day all-cause mortality, n (%)	5 (1.1)	3 (2.7)	0.399	5 (2.3)	3 (2.7)	1.000

Data are expressed as median (IQR) or number (percentage)

*ICU* intensive care unit; *IQR* interquartile range

### Relationship between the duration of low oxygen saturation and post-bronchoscopy respiratory adverse events

We performed a sensitivity analysis by including the duration of low oxygen saturation as a covariate. In the univariable analysis, the longer duration of low oxygen saturation was associated with increased risk of developing respiratory adverse events (odds ratio [OR] 1.02, 95% confidence interval [CI] 1.00–1.04, P = 0.047), and the multivariable analysis did not change the result (odds ratio [OR] 1.03, 95% confidence interval [CI] 1.00–1.05, P = 0.043) (Additional file 1: Table S6).

### Risk factors of post-bronchoscopy respiratory adverse events

We determined the clinical factors related to post-bronchoscopy respiratory adverse events (Additional file 1: Tables S1–S4). In the total population, older age, ever-smoking history, dementia, a lower percentage of predicted forced vital capacity, lower pre-bronchoscopy SF ratio, clinical suspicion of aspiration pneumonia, but low oxygen saturation during FB were risk factors for post-bronchoscopy respiratory adverse events (Additional file 1: Table S5). After adjustment, low oxygen saturation during FB was found to be an independent risk factor for post-bronchoscopy respiratory adverse events (odds ratio [OR] 2.45, 95% confidence interval [CI] 1.26–4.79, P = 0.009).

In the propensity score-matched population, older age, desaturation before FB, lower pre-bronchoscopy SF ratio, lack of a sedative agent, toilet bronchoscopy, and low oxygen saturation during FB were risk factors for post-bronchoscopy respiratory adverse events (Table 5). After adjustment, low oxygen saturation during FB was an independent risk factor for post-bronchoscopy respiratory adverse events (OR 3.16, 95% CI 1.37–7.30, P = 0.007).

**Table 5:** Risk factors of post-bronchoscopy respiratory adverse events in a propensity score-matched population

	Univariable analysis		Multivariable analysis	
	OR (95% CI)	P-value	OR (95% CI)	P-value
Age	1.06 (1.02–1.10)	0.001	1.03 (1.00–1.07)	0.046
Desaturation event before bronchoscopy	15.68 (6.37–38.64)	< 0.001	3.90 (1.28–11.89)	0.017
Pre-bronchoscopy SF ratio	0.98 (0.98–0.99)	< 0.001	0.99 (0.98–0.99)	< 0.001
Sedation	0.12 (0.05–0.32)	< 0.001	0.29 (0.09–0.92)	0.036
Bronchoscopic toileting	4.70 (2.46–8.96)	< 0.001	0.31 (0.10–1.02)	0.053
Bronchoscopic washing	0.32 (0.17–0.60)	< 0.001	0.36 (0.13–0.96)	0.042
Fellow physician, supervised by attending physician (reference: attending physician alone)	1.32 (0.70–2.47)	0.389	1.10 (0.49–2.46)	0.816
Low oxygen saturation group	2.16 (1.01–4.59)	0.046	3.16 (1.37–7.30)	0.007

*CI* confidence interval; *OR* odds ratio; *SF ratio* SpO_2_/FiO_2_ ratio

**Fig. 2:**
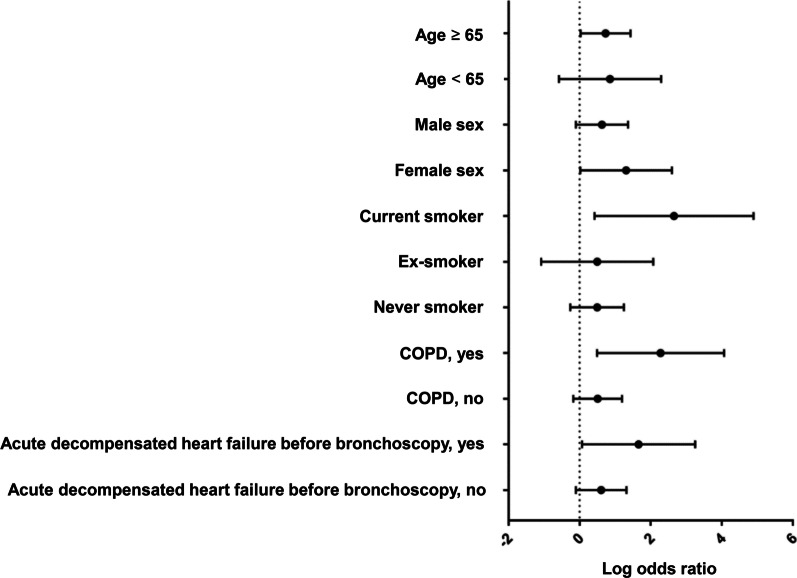
Association between low oxygen saturation during bronchoscopy and post-bronchoscopy respiratory adverse events in the different subgroups. *COPD* chronic obstructive pulmonary disease. The risk for post-bronchoscopy respiratory adverse events in the low oxygen saturation group was elevated in subgroups of patients older than 65 years, women, current smokers, diagnosed with COPD, and who presented with acute decompensated heart failure before FB

We evaluated the association between low oxygen saturation during FB and post-bronchoscopy respiratory adverse events in the different subgroups. The risk for post-bronchoscopy respiratory adverse events in the low oxygen saturation group was elevated in subgroups of patients older than 65 years, women, current smokers, diagnosed with COPD, and who presented with acute decompensated heart failure before FB (Fig. 2).

## Discussion

Our study investigated the association between post-bronchoscopy clinical outcomes and oxygen saturation levels during FB. There were more post-bronchoscopy respiratory adverse events in the low oxygen saturation group than in the high oxygen saturation group. In the logistic regression model, low oxygen saturation during FB was an independent risk factor for post-bronchoscopy respiratory adverse events even after adjusting for pre-existing lung conditions, such as dyspnea or SF ratio before FB. The subgroups at high risk for respiratory adverse events in the low oxygen saturation group were the elderly, women, current smokers, and patients with COPD or acute decompensated heart failure before FB.

Although post-bronchoscopy respiratory complications have been discussed in different study settings, there is no consensus on their definition [23]. In a systematic review that assessed post-bronchoscopy complications and discomfort, respiratory complications included pneumothorax, respiratory tract bleeding, bronchospasm, respiratory infection, and other symptoms (dyspnea, cough, and change in asthma symptom scores) that occurred within 2 weeks after bronchoscopy [23]. Severe respiratory complications include airway obstruction, tracheoesophageal fistula, tracheal perforation, and uncontrolled respiratory tract bleeding [24]. A case–control study evaluating the risk factors for post-bronchoscopy pneumonia defined bronchoscopy-related infection as occurring within 30 days after bronchoscopy, as per the surgical site infection guidelines issued by the Centers for Disease Control and Prevention. In our study, respiratory adverse events were defined as a composite outcome that included pneumonia, atelectasis, respiratory failure, pneumothorax, bronchospasm, acute exacerbation of an underlying chronic respiratory disease, and worsening of respiratory symptoms (dyspnea, cough, and purulent sputum), which occurred within 72 h after the procedure.

Post-bronchoscopy respiratory adverse events may delay the improvement of the primary lung condition or lead to fatal events [25]. Although FB is considered a safe procedure, it is invasive, and severe complications have been increasingly reported because of its wide use [24]. Previous studies have identified risk factors for post-bronchoscopy complications. For example, dyspnea requiring intervention has been reported in many patients with COPD or asthma after bronchoscopy [26]. In a study of 2,265 patients, post-bronchoscopy pneumonia developed more frequently in patients who had a smoking history or received BAL. Moreover, tracheobronchial stenosis was reported as an independent risk factor for post-bronchoscopy pneumonia [27]. In patients with lung cancer, old age, current smoking status, and central tumor location were independent predictors of post-bronchoscopy pneumonia [25].

The British thoracic society guideline for oxygen use in adults in health care and emergency settings recommends that oxygen be prescribed to achieve a target saturation of 94–98% for acutely ill patients [28]. Therefore, we defined the low oxygen saturation group as patients with SpO_2_ ≤ 94% during FB, below the suggested target oxygen saturation level. The definition of high oxygen saturation as > 96% was also based on literature regarding oxygenation strategies in adult patients. The liberal or conservative oxygen therapy (LOCO_2_) trial, which assessed the optimal oxygen therapy for acute respiratory distress syndrome, assigned patients with SpO_2_ targets above 96% to the liberal oxygen therapy group [29]. In the ICU-ROX trial, the upper limit of the SpO_2_ was 97% in the conservative-oxygen group [30]. We decided to exclude those with SpO_2_ between 94 and 96% to clearly delineate oxygen saturation between the two groups.

Our results revealed that post-bronchoscopy respiratory adverse events developed more frequently in the low oxygen saturation group. Nevertheless, there was no significant difference in clinical outcomes after FB between the two groups, suggesting that most respiratory events could be appropriately managed if detected. Therefore, close monitoring after FB may improve clinical outcomes in patients with low oxygen saturation during FB.

The increase in post-bronchoscopy respiratory adverse events in the low oxygen saturation group may be due to the pathophysiological impact of FB on cardiopulmonary distress. Hypoxemia in this group of patients subsequently triggers chained and amplified inflammatory responses. Bronchoscopy alters respiratory mechanics by increasing airflow resistance and work of breathing. These changes are detrimental to gas exchange and may take minutes to hours to revert [10]. Bronchoscopy also causes a significant acute decline in pulmonary function [31]. When suction is applied, the partial pressure of CO_2_ in arterial blood (PaCO_2_) rises to 30%, while the partial pressure of O_2_ in arterial blood (PaO_2_) decreases up to 40% due to reduced end-expiratory volume and positive end-expiratory pressure (PEEP) [10]. This leads to alveolar de-recruitment, increased shunt and venous admixture [10], and transient hypoxemia-related inflammatory cytokine recruitment, which aggravates inflammation in these hypoxic lung tissues. The hypoxia-inducible factor-prolyl hydroxylase (HIF-PHD) system exacerbates the inflammatory processes in the airway epithelial cells by inducing neutrophil chemotaxis and the release of reactive oxygen species, proteinases, and neutrophil extracellular traps, resulting in tissue damage [32]. Additionally, fluid accumulation in alveolar sacs and alveolar de-recruitment after FB promotes regional tissue hypoxia and inflammatory changes in the airway epithelial cells [33].

Moreover, low oxygen saturation during FB might represent an impaired cardiopulmonary reservoir incapable of adequate hemodynamic adaptations to hypoxemia [34]. In healthy individuals with adequate cardiopulmonary function, an acute hypoxemic condition causes compensatory responses, such as regional pulmonary vasoconstriction, hyperventilation, acidosis-related right shifting of the oxyhemoglobin dissociation curve, and an increase in cardiac output, effectively improving oxygenation to tissues [35–38]. However, insufficient compensatory mechanisms render fragile patients with marginal cardiopulmonary reservoir vulnerable to hypoxic stress, which activates a vicious cycle of inflammation and infection [39]. In support of our explanation, the elderly have shown a 50% reduction in protective pathophysiologic response to hypoxia and a 40% reduction in response to hypercapnia compared to young men [40]. Moreover, evidence of cardiac strain was observed in 21% of the patients aged > 60 years undergoing FB [41].

Oxygen supplementation provides sufficient oxygen to maintain normal physiologic levels during bronchoscopy, thus preventing desaturation. Preventive oxygen supplementation of 2–3 L/min may benefit patients at high risk of desaturation [42]. In patients with diffuse interstitial lung disease undergoing BAL or transbronchial lung biopsy, supplemental oxygen reduced significant hypoxemia events [1]. Continuous NIV support during bronchoscopy-guided nasal intubation prevents severe desaturation in critically ill patients [43]. In our study, 74% of patients in the low oxygen saturation group experienced desaturation events, and 11% experienced prolonged and significant (> 4% change or SpO_2_ < 90%) desaturation. Oxygen supplementation is strongly recommended in such cases [1]. Therefore, preventive oxygen supplementation in the low oxygen saturation group may reduce desaturation events and hypoxemia-related complications. Among the diverse oxygenation strategies, evidence is scarce about the advantages of one modality over another [5].

Our study has limitations. First, it was retrospective in nature, and the number of post-bronchoscopy complications may have been underestimated. Second, not all adverse events were directly triggered by FB, and comorbid lung conditions may have had a greater effect on the development of adverse events. Notably, the pre-bronchoscopy SF ratio, an indicator of hypoxemia severity, was a significant factor associated with post-bronchoscopy adverse events. Therefore, we evaluated various putative markers for respiratory failure before FB and conducted propensity score matching with comorbidities and severity of lung disease. Even in the matched study population, the oxygen saturation level during FB was significantly associated with post-bronchoscopy adverse events. Third, the patients with severely fluctuating SpO_2_ during FB were not included in our analyses. This study was designed to include the patients exposed solely to either one condition (SpO_2_ > 96% or SpO_2_ ≤ 94%) to diminish the potential confounding detrimental or beneficial effect of the other condition. Fourth, about 60% of our study population underwent bronchial washing, and 20% and 10% received toileting and BAL, respectively. As the diagnostic value, availability, and preference for bronchoscopic procedures may vary among institutions, caution is warranted against generalizing our results, especially in institutions that frequently perform more invasive procedures on unstable patients. Fifth, our study population mainly consisted of clinically stable patients undergoing pre-scheduled bronchoscopy. Accordingly, our findings may not apply to patients with more severe illnesses (e.g., patients admitted to the ICU). As the cardiopulmonary function of these patients is at a reduced capacity, the pathophysiological impact of hypoxemia during FB in this population should be investigated in the future. Sixth, the events in the outpatients were self-reported during a routine follow-up within 7 days after FB, which may lead to recall bias. However, the interval between the event and recall was sufficiently short. Additionally, all of the events and additional health care utilization were recorded on the electronic medical record (EMR), minimizing recall bias. Seventh, sampling bias may have affected the results. However, these were minimized by adjusting the propensity scores using the covariates in the patients’ baseline characteristics and clinical features. Finally, we did not perform further multivariable analysis by adjusting the covariates related to prolonged hospitalization, ICU admission, or 7-day all-cause mortality. Therefore, the prognosis or the medical impact of the adverse events should be further investigated through future studies.

## Conclusions

Low oxygen saturation level during FB may be an independent risk factor for post-bronchoscopy respiratory adverse events. An intensive monitoring system with preventive oxygen supplementation may benefit patients with low oxygen saturation levels during FB, especially in the high-risk subgroups.

## Supplementary Information

**Additional file 1: Table S1.** Baseline characteristics of total and propensity score-matched patients with respiratory adverse events. Data are expressed as mean (± standard deviation), median (IQR), or number (percentage). PYs, pack-years; SD, standard deviation; IQR, interquartile range. **Table S2.** Clinical features before bronchoscopy in total and propensity score-matched patients with respiratory adverse events. Data are expressed as mean (± standard deviation), median (IQR), or number (percentage). FVC, forced vital capacity; FEV1, forced expiratory volume in one second; BDR, bronchodilator test; DLCO, diffusion capacity of the lung for carbon monoxide; DLCO/VA, diffusion capacity of the lung for carbon monoxide per unit alveolar volume; SD, standard deviation; IQR, interquartile range. ^a^Bronchodilator response was assessed in 342 patients, and DLCO was assessed in 360 patients. **Table S3.** Parameters and procedures during bronchoscopy in patients with respiratory adverse events. Data are expressed as mean (± standard deviation) or number (percentage). EBUS-TBNA, endobronchial ultrasound-guided transbronchial needle aspiration; TBLB, transbronchial lung biopsy. **Table S4.** Post-bronchoscopy adverse events and clinical outcomes in patients with respiratory adverse events. Data are expressed as median (IQR) or number (percentage). ICU, intensive care unit; IQR, interquartile range. **Table S5.** Risk factors of post-bronchoscopy respiratory adverse events in the total population. CI, confidence interval; OR, odds ratio; SF ratio, SpO_2_/FiO_2_ ratio. Covariables were selected according to the rule of thumb. **Table S6.** Sensitivity analysis to evaluate the relationship between the duration of low oxygen saturation and post-bronchoscopy respiratory adverse events. CI, confidence interval; OR, odds ratio; SF ratio, SpO_2_/FiO_2_ ratio.

## Data Availability

The datasets used or analyzed during the current study are available from the corresponding author on reasonable request.

## Abbreviations

AMI: Acute myocardial infarction
BAL: Bronchoalveolar lavage
BMI: Body mass index
BDR: Bronchodilator test
CI: Confidence interval
COPD: Chronic obstructive pulmonary disease
CT: Computed tomography
DL_CO_: Diffusion capacity for the lung for carbon monoxide
DL_CO_/VA: Diffusion capacity for the lung for carbon monoxide per unit alveolar volume
EBUS-TNBA: Endobronchial ultrasound-guided transbronchial needle aspiration
ECG: Electrocardiography
EMR: Electronic medical record
FB: Flexible bronchoscopy
FEV1: Forced expiratory volume in one second
FVC: Forced vital capacity
ICU: Intensive care unit
ILD: Interstitial lung disease
IQR: Interquartile range
LOS: Length of stay
NIV: Noninvasive ventilation
OR: Odds ratio
PaCO_2_: Partial pressure of CO_2_ in arterial blood
PaO_2_: Partial pressure of O_2_ in arterial blood
PEEP: Positive end-expiratory pressure
HIF-PHD: Hypoxia-inducible factor-prolyl hydroxylase
PY: Pack-years
ROX: Respiratory rate-oxygenation
SF ratio: SpO_2_/FiO_2_ ratio
SD: Standard deviation
SpO_2_: Saturation of percutaneous oxygen
STROBE: Strengthening the reporting of observational studies in epidemiology
TBLB: Transbronchial lung biopsy
