# Supplementary material for: Association between oxygen saturation level during bronchoscopy and post-bronchoscopy adverse events: a retrospective cohort study
Source: Respir Res. 2022 Jun 2;23:144. doi: 10.1186/s12931-022-02063-0 (PMC9161191; doi:10.1186/s12931-022-02063-0)
Supplement: Supplementary file 1 — Additional file 1: Table S1. Baseline characteristics of total and propensity score-matched patients with respiratory adverse events. Data are expressed as mean (± standard deviation), median (IQR), or number (percentage). PYs, pack-years; SD, standard deviation; IQR, interquartile range. Table S2. Clinical features before bronchoscopy in total and propensity score-matched patients with respiratory adverse events. Data are expressed as mean (± standard deviation), median (IQR), or number (percentage). FVC, forced vital capacity; FEV1, forced expiratory volume in one second; BDR, bronchodilator test; DLCO, diffusion capacity of the lung for carbon monoxide; DLCO/VA, diffusion capacity of the lung for carbon monoxide per unit alveolar volume; SD, standard deviation; IQR, interquartile range. aBronchodilator response was assessed in 342 patients, and DLCO was assessed in 360 patients. Table S3. Parameters and procedures during bronchoscopy in patients with respiratory adverse events. Data are expressed as mean (± standard deviation) or number (percentage). EBUS-TBNA, endobronchial ultrasound-guided transbronchial needle aspiration; TBLB, transbronchial lung biopsy. Table S4. Post-bronchoscopy adverse events and clinical outcomes in patients with respiratory adverse events. Data are expressed as median (IQR) or number (percentage). ICU, intensive care unit; IQR, interquartile range. Table S5. Risk factors of post-bronchoscopy respiratory adverse events in the total population. CI, confidence interval; OR, odds ratio; SF ratio, SpO2/FiO2 ratio. Covariables were selected according to the rule of thumb. Table S6. Sensitivity analysis to evaluate the relationship between the duration of low oxygen saturation and post-bronchoscopy respiratory adverse events. CI, confidence interval; OR, odds ratio; SF ratio, SpO2/FiO2 ratio. [file 12931_2022_2063_MOESM1_ESM.docx]

**Additional file 1**

**Table S1. Baseline characteristics of total and propensity score-matched patients with respiratory adverse events**

|  | | **Total population** | | | | | | **Propensity score-matched population** | | | | | |
| --- | --- | --- | --- | --- | --- | --- | --- | --- | --- | --- | --- | --- | --- |
|  |  | **With respiratory adverse events (n = 66)** | **Without respiratory adverse events (n = 503)** | | **P-value** | | **With respiratory adverse events (n = 47)** | | | **Without respiratory adverse events (n = 286)** | | **P-value** | |
| **Age, yr, mean (±SD)** | | 72.9 (14.2) | 66.2 (13.9) | | < 0.001 | | 74.9 (13.6) | | | 65.3 (14.8) | | < 0.001 | |
| **Female, n (%)** | | 15 (22.7) | 174 (34.6) | | 0.074 | | 11 (23.4) | | | 112 (39.2) | | 0.056 | |
| **Body mass index, mean (±SD)** | | 20.7 (4.9) | 21.6 (4.2) | | 0.108 | | 21.5 (5.2) | | | 22.2 (4.5) | | 0.337 | |
| **Smoking status** | |  |  | |  | |  | | |  | |  | |
| Current smoker, n (%) | | 6 (9.1) | 98 (19.5) | | 0.059 | | 6 (12.8) | | | 50 (17.5) | | 0.555 | |
| Ex-smoker, n (%) | | 14 (21.2) | 135 (26.8) | | 0.407 | | 7 (14.9) | | | 70 (24.5) | | 0.209 | |
| Never smoker, n (%) | | 46 (69.7) | 262 (52.1) | | 0.010 | | 34 (72.3) | | | 163 (57.0) | | 0.068 | |
| PYs, median (IQR) | | 0 (0–15) | 0 (0–30) | | 0.034 | | 0 (0–10) | | | 0 (0–20) | | 0.111 | |
| **Comorbidities, n (%)** | |  |  | |  | |  | | |  | |  | |
| Hypertension | | 28 (42.4) | 197 (39.2) | | 0.707 | | 21 (44.7) | | | 108 (37.8) | | 0.459 | |
| Diabetes mellitus | | 18 (27.3) | 134 (26.6) | | 1.000 | | 13 (27.7) | | | 62 (21.7) | | 0.471 | |
| Congestive heart failure | | 15 (22.7) | 55 (10.9) | | 0.011 | | 12 (25.5) | | | 39 (13.6) | | 0.060 | |
| Arrhythmia | | 4 (6.1) | 20 (4.0) | | 0.641 | | 3 (6.4) | | | 13 (4.5) | | 0.859 | |
| Cerebrovascular accident | | 17 (25.8) | 86 (17.1) | | 0.122 | | 13 (27.7) | | | 34 (11.9) | | 0.008 | |
| Chronic kidney disease | | 16 (24.2) | 26 (5.2) | | < 0.001 | | 11 (23.4) | | | 12 (4.2) | | < 0.001 | |
| Chronic liver disease | | 3 (4.5) | 31 (6.2) | | 0.806 | | 3 (6.4) | | | 15 (5.2) | | 1.000 | |
| Connective tissue disease | | 2 (3.0) | 14 (2.8) | | 1.000 | | 2 (4.3) | | | 6 (2.1) | | 0.703 | |
| Dementia | | 15 (22.7) | 33 (6.6) | | < 0.001 | | 9 (19.1) | | | 17 (5.9) | | 0.005 | |
| History of other malignancy | | 18 (27.3) | 85 (16.9) | | 0.059 | | 10 (21.3) | | | 50 (17.5) | | 0.673 | |
| **Respiratory disease, n (%)** |  | | |  | |  | | |  | |  | |  |
| Chronic obstructive pulmonary disease | | 11 (16.7) | 84 (16.7) | | 1.000 | | 8 (17.0) | | | 34 (11.9) | | 0.456 | |
| Bronchiectasis | | 13 (19.7) | 148 (29.4) | | 0.133 | | 11 (23.4) | | | 71 (24.8) | | 0.979 | |
| Asthma | | 3 (4.5) | 15 (3.0) | | 0.758 | | 2 (4.3) | | | 11 (3.8) | | 1.000 | |
| Interstitial lung disease | | 1 (1.5) | 15 (3.0) | | 0.778 | | 1 (2.1) | | | 7 (2.4) | | 1.000 | |
| Tuberculosis destroyed lung | | 17 (25.8) | 76 (15.1) | | 0.043 | | 9 (19.1) | | | 22 (7.7) | | 0.025 | |
| Nontuberculous mycobacteria | | 0 (0.0) | 19 (3.8) | | 0.214 | | 0 (0.0) | | | 12 (4.2) | | 0.313 | |
| History of lung cancer | | 3 (4.5) | 36 (7.2) | | 0.596 | | 3 (6.4) | | | 20 (7.0) | | 1.000 | |
| History of lung resection | | 0 (0.0) | 15 (3.0) | | 0.311 | | 0 (0.0) | | | 10 (3.5) | | 0.401 | |
| History of thoracic radiation therapy | | 3 (4.5) | 13 (2.6) | | 0.610 | | 3 (6.4) | | | 5 (1.7) | | 0.159 | |

Data are expressed as mean (± standard deviation), median (IQR), or number (percentage).

PYs, pack-years; SD, standard deviation; IQR, interquartile range

**Table S2. Clinical features before bronchoscopy in total and propensity score-matched patients with respiratory adverse events**

|  | **Total population** | | | | **Propensity score-matched population** | | |
| --- | --- | --- | --- | --- | --- | --- | --- |
|  | **With respiratory adverse events (n = 66)** | **Without respiratory adverse events (n = 503)** | **P-value** | **With respiratory adverse events (n = 47)** | | **Without respiratory adverse events (n = 286)** | **P-value** |
| **Symptoms, n (%)** | | | | | | | |
| Chronic bronchitis | 16 (24.2) | 76 (15.1) | 0.086 | 6 (12.8) | | 35 (12.2) | 1.000 |
| Chronic cough | 7 (10.6) | 94 (18.7) | 0.149 | 3 (6.4) | | 52 (18.2) | 0.071 |
| Hemoptysis | 4 (6.1) | 61 (12.1) | 0.211 | 4 (8.5) | | 31 (10.8) | 0.821 |
| **Pulmonary function test^a^** | | | | | | | |
| FVC, L, mean (±SD) | 2.8 (0.3) | 2.8 (0.6) | 0.571 | 2.8 (0.3) | | 2.9 (0.6) | 0.511 |
| FVC, %, mean (±SD) | 81.8 (10.4) | 86.1 (13.8) | 0.014 | 84.0 (7.3) | | 88.4 (12.7) | 0.024 |
| FEV_1_, L, mean (±SD) | 2.1 (0.4) | 2.1 (0.5) | 0.710 | 2.05 (0.3) | | 2.1 (0.5) | 0.635 |
| FEV_1_, %, mean (±SD) | 87.0 (10.1) | 90.2 (17.4) | 0.138 | 88.9 (6.2) | | 93.0 (16.4) | 0.098 |
| FEV_1_/FVC, %, mean (±SD) | 75.5 (7.7) | 73.4 (8.9) | 0.063 | 74.3 (5.4) | | 73.7 (8.1) | 0.633 |
| BDR, %, median (IQR) | 2.0 (1.0–4.0) | 2.0 (-1.0–3.0) | 0.302 | 1.5 (-6.0–4.0) | | 2.0 (0–3.0) | 0.580 |
| DL_CO_, %, mean (±SD) | 73.4 (25.9) | 89.7 (28.6) | 0.024 | 80.9 (29.9) | | 92.9 (25.2) | 0.156 |
| DL_CO_/VA, %, mean (±SD) | 64.7 (31.3) | 88.6 (26.4) | 0.001 | 72.9 (24.3) | | 92.8 (22.9) | 0.010 |
| **Clinically suspected diagnosis, n (%)** | | | | | | | |
| Aspiration pneumonia | 120 (26.2) | 28 (25.2) | 0.929 | 59 (26.6) | | 28 (25.2) | 0.895 |
| Atypical pneumonia | 41 (9.0) | 18 (16.2) | 0.038 | 21 (9.5) | | 18 (16.2) | 0.104 |
| Lung malignancy | 121 (26.4) | 30 (27.0) | 0.992 | 59 (26.6) | | 30 (27.0) | 1.000 |
| Mycobacterial infection | 176 (38.4) | 35 (31.5) | 0.215 | 81 (36.5) | | 35 (31.5) | 0.440 |
| Lung abscess | 21 (4.6) | 5 (4.5) | 1.000 | 10 (4.5) | | 5 (4.5) | 1.000 |
| Interstitial lung disease | 22 (4.8) | 7 (6.3) | 0.685 | 13 (5.9) | | 7 (6.3) | 1.000 |
| Endobronchial lesion | 111 (24.2) | 16 (14.4) | 0.036 | 45 (20.3) | | 16 (14.4) | 0.249 |
| **Combined medical conditions, n (%)** | | | | | | | |
| Acute decompensated heart failure | 13 (19.7) | 33 (6.6) | 0.001 | 12 (25.5) | | 19 (6.6) | < 0.001 |
| Acute coronary syndrome | 5 (7.6) | 8 (1.6) | 0.009 | 5 (10.6) | | 7 (2.4) | 0.018 |
| Pneumothorax | 1 (1.5) | 17 (3.4) | 0.66 | 0 (0.0) | | 8 (2.8) | 0.518 |
| Pleural effusion | 39 (59.1) | 122 (24.3) | <0.001 | 26 (55.3) | | 63 (22.0) | < 0.001 |
| **Surrogates for respiratory failure** | | | | | | | |
| Desaturation event, n (%) | 25 (37.9) | 144 (28.6) | 0.161 | 22 (46.8) | | 106 (37.1) | 0.267 |
| Oxygen demand, L/min, median (IQR) | 1 (0–3.0) | 0 (0–0) | < 0.001 | 2.0 (1.0–4.0) | | 0 (0–0) | < 0.001 |
| SF ratio, mean (±SD) | 375.3 (93.2) | 455.9 (42.7) | < 0.001 | 345.6 (93.7) | | 449.8 (49.1) | < 0.001 |
| ROX index, mean (±SD) | 19.2 (5.2) | 22.9 (2.1) | < 0.001 | 17.8 (5.4) | | 22.6 (2.3) | < 0.001 |

Data are expressed as mean (± standard deviation), median (IQR), or number (percentage).

FVC, forced vital capacity; FEV_1_, forced expiratory volume in one second; BDR, bronchodilator test; DL_CO_, diffusion capacity of the lung for carbon monoxide; DL_CO_/VA, diffusion capacity of the lung for carbon monoxide per unit alveolar volume; SD, standard deviation; IQR, interquartile range

^a^Bronchodilator response was assessed in 342 patients, and DL_CO_ was assessed in 360 patients.

**Table S3. Parameters and procedures during bronchoscopy in patients with respiratory adverse events**

|  | **Total population** | | | | **Propensity score-matched population** | | |
| --- | --- | --- | --- | --- | --- | --- | --- |
|  | **With respiratory adverse events (n = 66)** | **Without respiratory adverse events (n = 503)** | **P-value** | **With respiratory adverse events (n = 47)** | | **Without respiratory adverse events (n = 286)** | **P-value** |
| **Oxygen saturation during bronchoscopy** | | | | | | | |
| Initial SpO_2_, mean (±SD) | 98.1 (2.3) | 98.6 (2.0) | 0.037 | 97.7 (2.6) | | 98.3 (2.2) | 0.130 |
| Highest SpO_2_, mean (±SD) | 97.6 (2.5) | 98.3 (2.2) | 0.010 | 96.9 (2.7) | | 97.7 (2.6) | 0.065 |
| Lowest SpO_2_, mean (±SD) | 92.5 (7.3) | 93.9 (6.1) | 0.079 | 90.8 (7.7) | | 92.4 (6.7) | 0.141 |
| Desaturation event, n (%) | 25 (37.9) | 144 (28.6) | 0.161 | 22 (46.8) | | 106 (37.1) | 0.267 |
| Desaturation duration >1 min, n, (%) | 3 (4.5) | 9 (1.8) | 0.313 | 3 (6.4) | | 9 (3.1) | 0.496 |
| **Sedation, n (%)** | 6 (9.1) | 171 (34.0) | < 0.001 | 5 (10.6) | | 141 (49.3) | < 0.001 |
| **Procedure type, n (%)** |  |  |  |  | |  |  |
| Bronchial washing | 25 (37.9) | 346 (68.8) | < 0.001 | 18 (38.3) | | 189 (66.1) | 0.001 |
| Toileting | 38 (57.6) | 95 (18.9) | < 0.001 | 24 (51.1) | | 52 (18.2) | < 0.001 |
| Bronchoalveolar lavage | 6 (9.1) | 37 (7.4) | 0.800 | 6 (12.8) | | 23 (8.0) | 0.432 |
| Biopsy | 4 (6.1) | 52 (10.3) | 0.380 | 4 (8.5) | | 27 (9.4) | 1.000 |
| EBUS-TBNA | 3 (4.5) | 52 (10.3) | 0.202 | 3 (6.4) | | 39 (13.6) | 0.250 |
| TBLB | 0 (0) | 2 (0.4) | 1.000 | 0 (0) | | 1 (0.3) | 1.000 |
| Foreign body removal | 9 (13.6) | 8 (1.6) | < 0.001 | 4 (8.5) | | 6 (2.1) | 0.054 |

Data are expressed as mean (± standard deviation) or number (percentage).

EBUS-TBNA, endobronchial ultrasound-guided transbronchial needle aspiration; TBLB, transbronchial lung biopsy

**Table S4. Post-bronchoscopy adverse events and clinical outcomes in patients with respiratory adverse events**

|  | **Total population** | | | | **Propensity score-matched population** | | |
| --- | --- | --- | --- | --- | --- | --- | --- |
|  | **With respiratory adverse events (n = 66)** | **Without respiratory adverse events (n = 503)** | **P-value** | **With respiratory adverse events (n = 47)** | | **Without respiratory adverse events (n = 286)** | **P-value** |
| **Post-bronchoscopy adverse events, n (%)** | | | | | | | |
| Febrile events | 20 (30.3) | 57 (11.3) | < 0.001 | 18 (38.3) | | 35 (12.2) | < 0.001 |
| Hemodynamic events | 25 (37.9) | 15 (3.0) | < 0.001 | 19 (40.4) | | 4 (1.4) | < 0.001 |
| Cardiac events | 10 (15.2) | 7 (1.4) | < 0.001 | 10 (21.3) | | 6 (2.1) | < 0.001 |
| Cerebrovascular events | 1 (1.5) | 0 (0.0) | 0.116 | 1 (2.1) | | 0 (0.0) | 0.141 |
| **Clinical outcomes** |  |  |  |  | |  |  |
| Hospital length of stay after bronchoscopy, median (IQR) | 19 (9-41) | 1 (1-9) | < 0.001 | 16 (9-36) | | 1 (1-8) | < 0.001 |
| ICU admission after bronchoscopy, n (%) | 19 (28.8) | 8 (1.6) | < 0.001 | 14 (29.8) | | 3 (1.0) | < 0.001 |
| 7-day all-cause mortality, n (%) | 4 (6.1) | 4 (0.8) | 0.004 | 4 (8.5) | | 4 (1.4) | 0.015 |

Data are expressed as median (IQR) or number (percentage).

ICU, intensive care unit; IQR, interquartile range

**Table S5. Risk factors of post-bronchoscopy respiratory adverse events in the total population**

|  | **Univariable analysis** | | **Multivariable analysis** | |
| --- | --- | --- | --- | --- |
|  | **OR (95% CI)** | **P-value** | **OR (95% CI)** | **P-value** |
| Age | 1.04 (1.02-1.07) | < 0.001 | 1.01 (0.98-1.03) | 0.588 |
| Ever-smoker | 2.87 (1.19-6.93) | < 0.001 | 2.75 (1.00-7.54) | 0.049 |
| Dementia | 4.19 (2.13-8.23) | < 0.001 | 2.59 (1.11-6.07) | 0.028 |
| FVC, % of predicted value | 0.98 (0.96-1.00) | 0.014 | 0.99 (0.97-1.01) | 0.210 |
| Pre-bronchoscopy SF ratio | 0.98 (0.98-0.99) | < 0.001 | 0.99 (0.98-0.99) | < 0.001 |
| Aspiration pneumonia | 5.22 (3.06-8.91) | < 0.001 | 2.17 (1.12-4.20) | 0.022 |
| Low-oxygen saturation group | 2.52 (1.45-4.40) | 0.001 | 2.45 (1.26-4.79) | 0.009 |

CI, confidence interval; OR, odds ratio; SF ratio, SpO_2_/FiO_2_ ratio

Covariables were selected according to the rule of thumb.

**Table S6. Sensitivity analysis to evaluate the relationship between the duration of low oxygen saturation and post-bronchoscopy respiratory adverse events**

|  | **Univariable analysis** | | **Multivariable analysis** | |
| --- | --- | --- | --- | --- |
|  | **OR (95% CI)** | **P-value** | **OR (95% CI)** | **P-value** |
| Age | 1.06 (1.02–1.10) | 0.001 | 1.01 (0.99–1.04) | 0.331 |
| Desaturation event before bronchoscopy | 15.68 (6.37–38.64) | < 0.001 | 5.49 (2.55–11.85) | < 0.001 |
| Pre-bronchoscopy SF ratio | 0.98 (0.98–0.99) | < 0.001 | 0.9923 (0.9875–0.997) | 0.001 |
| Duration of low oxygen saturation in min | 1.02 (1.00–1.04) | 0.047 | 1.03 (1.00–1.05) | 0.043 |

CI, confidence interval; OR, odds ratio; SF ratio, SpO_2_/FiO_2_ ratio
